# Low effectiveness of syndromic treatment services for curable sexually transmitted infections in rural South Africa

**DOI:** 10.1136/sti.2008.032011

**Published:** 2008-08-15

**Authors:** R G White, P Moodley, N McGrath, V Hosegood, B Zaba, K Herbst, M Newell, W A Sturm, R J Hayes

**Affiliations:** 1 Department of Epidemiology and Population Health, London School of Hygiene and Tropical Medicine, London, UK; 2 Department of Medical Microbiology, Nelson R. Mandela School of Medicine, University of KwaZulu-Natal, South Africa; 3 Department of Infection Control, Nelson R Mandela School of Medicine, University of KwaZulu-Natal, South Africa; 4 Africa Centre for Health and Population Studies, University of KwaZulu-Natal, Somkhele, South Africa

## Abstract

**Objectives:**

Syndromic sexually transmitted infection (STI) treatment remains a cost-saving HIV prevention intervention in many countries in Africa. We estimate the effectiveness of syndromic treatment for curable STIs in rural KwaZulu-Natal, South Africa, and the trend in STI prevalences before and after the introduction of syndromic treatment in 1995.

**Methods:**

Data were available from various clinical studies, surveys of public and private health providers, the general population and women attending antenatal, family planning and child immunisation clinics in rural northern KwaZulu-Natal between 1987 and 2004. Overall effectiveness was defined as the estimated proportion of the annual number of symptomatic curable STI episodes cured by syndromic treatment based on separate estimates for six curable STI aetiologies by gender.

**Results:**

Median overall effectiveness was 13.1% (95% CI 8.9 to 17.8%) of symptomatic curable STI episodes cured. Effectiveness increased to 25.0% (95% CI 17.3 to 33.8%), 47.6% (95% CI 44.5 to 50.8%) or 14.3% (95% CI 9.9 to 19.4%) if 100% treatment seeking, 100% correct treatment provision or 100% cure were assumed, respectively. Time-trends were difficult to assess formally but there was little evidence of decreasing STI prevalences. Including incurable but treatable herpes simplex virus (HSV)-2 ulcers in the effectiveness calculation would halve the proportion of ulcers cured or correctly treated, but this reduction could be entirely countered by including episodic antiviral treatment in the national guidelines.

**Conclusion:**

Overall effectiveness of syndromic treatment for curable STIs in rural KwaZulu-Natal remains low and there is little evidence of reduced curable STI prevalences. As syndromic treatment is likely to be a cost-saving HIV prevention intervention in South Africa, innovative strategies are urgently needed to increase rates of treatment seeking and correct treatment provision.

HIV continues to spread throughout sub-Saharan Africa.^1^ Interventions to reduce the transmission of HIV include behaviour change and reduction of the per-sex-act transmission probability of HIV. Among the latter, syndromic case management of sexually transmitted infections (STIs) is important and widely implemented, and although the relative impact of syndromic STI treatment on HIV incidence is likely to fall over the course of HIV epidemics,^2^ it may remain a cost-saving intervention in many countries in sub-Saharan Africa, particularly those with high HIV incidence.^3^

South Africa has continuing high rates of STIs and HIV.^4^ ^5^ STI treatment services are provided by the public and private sectors. In the public sector, syndromic sexually transmitted disease (STD) case management is recommended based on the 1994 World Health Organization (WHO) guidelines, adapted and implemented since 1995 by the South African Department for Health.^6^ In the private sector, STI management is at the discretion of the individual general practitioner (GP).^7^ Antenatal clinic attendees are also screened and treated for syphilis.^8^

We estimate the effectiveness of the syndromic STI treatment services for curable STIs in rural KwaZulu-Natal, South Africa, on the basis of various sources of information and describe the trend in curable STI prevalences from before the introduction of syndromic STI management in 1995 until 2004.

## METHODS

### Data

Unless otherwise noted, data collection was restricted to the population in the southeast of Hlabisa sub-district, rural KwaZulu-Natal. The proportion of STI episodes with recognised clinical signs (“symptomatic episodes”) for which treatment was sought among males and females aged 15–49 years old was estimated from primary analysis of self-reported data collected in a general population survey in 2003–4 in the southeast part of the Umkhanyakude district, formerly part of the Hlabisa sub-district.^5^ The data presented here are on individuals whose primary place of residence was one of the households within the surveillance area (“residents”). Consenting adults, who reported genital ulcers or abnormal genital discharge in the preceding 12 months, were asked whether they had sought treatment from a doctor or clinic. Further, the proportion of patients seeking treatment for STI symptoms for whom effective treatment was prescribed was estimated from another surveillance study and two studies of prescribing patterns of public and private providers in Hlabisa sub-district in the late 1990s.^7^ ^9^ ^10^ The proportion cured among those given appropriate treatment was measured in a series of clinical studies carried out in facilities in the Hlabisa sub-district in 1999/2000^11^ and 2003,^12^ and Durban in 2000/2001.^13^ ^14^ The proportions cured were estimated from patients with a single microbiologically identified and curable aetiology at baseline and will reflect both the clinical efficacy of the treatment and patient adherence.

Data on prevalence of curable STIs were available from review of data collected in surveys of the general population and women attending antenatal, family planning and child immunisation clinics in the Hlabisa sub-district between 1987 and 2004.^15^^–^21 Syphilis prevalence trend data (diagnosed using rapid plasma reagin (RPR) only) were available from the Department of Health.^22^ Bias in STI prevalence trend data due to sample differences was limited by restricting data to the populations listed above.^15^^–^21 Data were unadjusted for STI diagnostic differences.

### Effectiveness calculation

The effectiveness of syndromic STI treatment was defined as


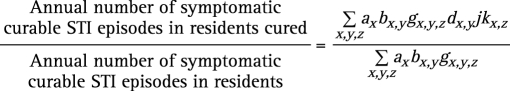


Where *x* is gender, *y* is syndrome and *z* is STI aetiology; *a_x_* is the number of residents aged 15–49 years of gender *x*; *b_x,y_* is the proportion of residents of gender *x* reporting syndrome *y* in the past year; *g_x,y,z_* is the proportion of clinic patients of gender *x* with syndrome *y* who are infected with curable aetiology *z*; *d_x,y_* is the proportion of symptomatic STI episodes for which treatment was sought for gender *x* and syndrome *y*; *j* is the proportion of patients seeking treatment who were given appropriate treatment and *k_x,z_* is the proportion cured among those given appropriate treatment for gender *x* and STI aetiology *z*. The overall effectiveness estimate was based on separate estimates for six curable STI aetiologies by gender: *Neisseria gonorrhoeae, Chlamydia trachomatis, C trachomatis (Lympho-granuloma venereum), Trichomonas vaginalis, Treponema pallidum* and *Haemophilus ducreyi*.

### Statistical analysis

Exact 95% CI for all proportions were based on the binomial distribution. In the effectiveness analysis, uncertainty in all parameter values was accounted for through specified beta distributions. Expected median values and 95% CIs for all derived parameters were calculated using Monte-Carlo sampling from the beta distributions using WinBUGS version 1.4.1.^23^ Results were based on 100 000 samples. The distribution specification and WinBUGS code are shown in the supplementary material.

### Sensitivity analysis

We analysed the robustness of our findings by re-calculating the effectiveness using alternative assumptions as detailed in full in the supplementary material.

## RESULTS

Altogether, 5424 males and 11 339 females aged 15–49 years old (44% and 70% of the resident population in these age/sex groups) participated in a sexual behaviour survey between January 2003 and August 2004.^5^ Although there were small statistically significant differences in some characteristics between the surveyed and not-surveyed groups, there was little suggestion of selection bias except that the survey over-sampled men 15–24 years old (survey = 61%, residents = 54%) and these men were more likely to have never married. Among individuals reporting ever having sex, 71% (2547/3587) of males and 69% (5897/8546) of females provided answers to questions about STI symptoms in the past year.

Discharge was reported by 6.8% (95% CI 6.1 to 7.6%; n/N = 299/4372) of males and 14.9% (95% CI 14.2 to 15.7%; 1288/8617) of females in the preceding year (*b*, table 1). Ulcers were reported by 4.3% (95% CI 3.7 to 4.9%; 188/4373) of males and 9.8% (95% CI 9.2 to 10.5%; 851/8,647) of females. Of those for which treatment seeking data were available and who reported a single syndrome in the period, 55.9% (95% CI 47.4 to 64.2%; 80/143) of males and 57.1% (95% CI 53.2 to 60.9%; 374/655) of females reported having sought treatment for discharge and 53.2% (95% CI 38.1 to 67.9%; 25/47) of males and 36.3% (95% CI 30.7 to 42.2%; 103/284) of females reported having sought treatment from a doctor or clinic for ulcers (*d*, table 1).

**Table 1:** Estimated overall effectiveness of syndromic treatment services

		No. of residents, 15–49 y	Proportion of survey population reporting syndrome in past year, 15–49 y	No. of residents reporting syndrome in past year, 15–49 y	Proportion of survey population who sought treatment at clinics, %	No. of residents seeking treatment at clinics	Syndrome aetiology	Proportion of clinic patients with aetiology	Proportion of clinic patients with curable aetiology	No. of symptomatic curable STIs in residents	No. of symptomatic curable STIs seen at clinics	Correct treatment, %*	Proportion cured, %	No. of symptomatic curable episodes due to this aetiology, cured, %	
Syndrome	Gender	*a*	*b*	c = a*b	*d*	e = c*d		*f*	*g*	h = c*g	i = e*g	*j*	*k*	l = i*j*k	*m = l/h*
GDS	M	12 250	6.8%	837.8	55.9	469	NG	0.59	0.59	498.1	278.7	27.4	100	76.4	15.2 (10.2, 21.2)
							CT	0.07	0.07	56.6	31.7	27.4	83	7.2	12.3 (7.8, 17.9)
							Other†	0.37							
	F	16 174	14.9%	2417.6	57.1	1380	NG	0.12	0.12	300.4	171.6	27.4	100	47.0	15.5 (10.6, 21.2)
							CT	0.11	0.11	255.0	145.6	27.4	87	34.7	13.4 (9.0, 18.5)
							TV	0.29	0.29	709.2	404.9	27.4	87	96.5	13.6 (9.3, 18.6)
							Other†	0.94							
All curable GDS															14.3 (9.8, 19.5)
GUD	M	12 250	4.3%	526.6	53.2	280	TP	0.16	0.16	86.6	46.0	27.4	89	11.2	12.5 (7.6, 18.9)
							HD	0.11	0.11	57.7	30.7	27.4	93	7.8	13.1 (8.1, 19.6)
							CT (LGV)	0.08	0.08	44.5	23.7	27.4	93	6.0	13.0 (8.0, 19.6)
							HSV-2	0.48							
							Other†	0.26							
	F	16 174	9.8%	1591.8	36.3	577	TP	0.09	0.09	138.9	50.4	27.4	89	12.3	8.6 (5.6, 12.4)
							HD	0.06	0.06	96.1	34.9	27.4	93	8.9	9.0 (5.9, 12.8)
							CT (LGV)	0.17	0.17	277.8	100.7	27.4	93	25.7	9.0 (5.9, 12.8)
							HSV-2	0.50							
							Other†	0.28							
All curable GUD															9.9 (6.6, 14.0)
Overall										2521.0	1318.9				13.1 (8.9, 17.8)

*Median (and 95% CI if applicable) calculated using Monte-Carlo sampling, see methods; †included other known and unknown aetiologies.

CT, *Chlamydia trachomatis*; F, female; GDS, genital discharge syndrome; GUD, genital ulcer disease; HD, *Haemophilus ducreyi*; HSV-2, herpes simplex virus type 2; LGV, *Lympho-granuloma venereum*; M, male; NG, *Neisseria gonorrhoeae*; TP, *Treponema pallidum*; TV, *Trichomonas vaginalis*; y, years.

Using the data sources above, we estimated 2521 symptomatic curable STIs in residents of which 52% (1319) were seen by GPs or at public clinics (*h* and *i*, table 1).

All public primary healthcare clinics and 8 of 12 GPs in the sub-district participated in the clinic-based surveillance study. Adjusting for the under-representation of GPs, of those seeking treatment for STI symptoms, 54% (95% CI 51 to 57%; 568/1052) of the STI episodes were treated in clinics and 46% (95% CI 43 to 49%; 484/1052) by GPs.^7^ This study was considered to be largely representative because all clinics and 67% of GPs participated. In two further studies of prescribing patterns in the public and private sectors for patients seeking treatment for STI syndromes, 41% (95% CI 26 to 57%; 18/44) of patients attending public clinics^10^ and 9% (95% CI 2 to 24%; 3/33) of patients attending their GPs^9^ received the treatment recommended by the Provincial Health Department. Using these data, we estimate that 27.4% (95% CI 18.9 to 37.1%) of patients who sought treatment for STI symptoms from public clinics or GPs received the correct treatment (*j*, table 1).

Table 2 shows data on the proportion cured among STI patients in KwaZulu-Natal treated using the current first-line treatment regimens from the national syndromic STI treatment guidelines.^6^ As the microbiological proportion cured for ulcerative STIs was not available (sampling disrupts epithelialisation), they were assumed equal to the clinical proportion cured. The proportion cured for males and females with gonorrhoea was assumed to be 100% despite increasing resistance to ciprofloxacin;^24^ this is in line with the data suggesting that gonorrhoea remains susceptible to ceftriaxone.^25^ The proportion cured for chlamydial and trichomonas infection in women were assumed to be the weighted mean from the two available studies as these were not significantly different (87%, 47/54 for *C trachomatis*; 87%,137/157 for *T vaginalis*).

**Table 2:** First-line treatment regimens and clinical and microbiological cure rates for national syndromic treatment guidelines by syndrome

Syndrome	Treatment regimen	Curable STI aetiology	n	Percentage cured		Reference
				Clinical % (n)	Microbiological % (n)	
Urethritis	500 mg single dose ciprofloxacin since 2001 (250 mg before 2001) and 200 mg of doxycycline daily for 7 days	NG	135	100 (135)	100 (135)	13
		CT	24	100 (24)	83 (20)	
Ulcer	One intramuscular injection of 2.4 million units of penicillin and 500 mg dose of erythromycin 4 times daily for 5 days.	TP	28	89 (25)		14
	No treatment for HSV-2	HD	30	93 (28)		
		CT (LGV)	29	93 (27)		
Vaginal discharge	500 mg single dose ciprofloxacin and 100 mg of doxycycline 2× daily for 7 days and 2000 mg metronidazole single dose, or 400 mg metronidazole 2× daily for 7 days	NG	51	65 (33)	80 (41)	11
		CT	44	69 (30)	89 (39)	
	Or if pregnant: one intramuscular injection of 125 mg ceftriaxone and erythromycin 500 mg 4× daily for 7 days and 400 mg metronidazole 2× daily for 7 days	TV	113	65 (73)	88 (98)	
		NG	11		100 (11)	12
	Or if cervical excitation tenderness: 500 mg single dose ciprofloxacin and 100 mg of doxycycline 2× daily for 14 days, and 400 mg metronidazole 2× daily for 14 days	CT	10		80 (8)	
		TV	44		89 (39)	

CT, *Chlamydia trachomatis*; HD, *Haemophilus ducreyi*; HSV-2, herpes simplex virus type 2; LGV, *Lympho-granuloma venereum*; NG, *Neisseria gonorrhoeae*; TP, *Treponema pallidum*; TV, *Trichomonas vaginalis*. Guidelines adapted from.^6^

### Estimated overall effectiveness

Based on these data, effectiveness by gender and aetiology ranged between 8.6% (95% CI 5.6 to 12.4%) for syphilis ulcers in females to 15.5% (95% CI 10.6 to 21.2%) for gonorrhoea in females (table 1). The overall effectiveness of the syndromic STI treatment services in rural KwaZulu-Natal was estimated to be 13.1% (95% CI 8.9 to 17.8%) of symptomatic curable STI episodes cured, 14.3% (95% CI 9.8 to 19.5%) for discharge and 9.9% (95% CI 6.6 to 14.0%) for ulcers.

### Sensitivity analysis

If 100% treatment seeking was assumed, the estimated overall effectiveness doubled to 25.0% (95% CI 17.3 to 33.8%). If 100% correct treatment provision was assumed, the overall effectiveness quadrupled to 47.6% (95% CI 44.5 to 50.8%). If 100% cure was assumed, the overall effectiveness increased slightly to 14.3% (95% CI 9.9 to 19.4%). If we assumed 10% of individuals who reported treatment at clinics or GPs actually went to traditional healers, the overall effectiveness fell slightly to 11.7% (95% CI 8.1 to 16.0%). If we assumed cure rates were overestimated by 25%, overall effectiveness decreased to 9.8% (95% CI 6.7 to 13.4%). If treatment seeking rates were 25% lower in survey non-participants, overall effectiveness fell slightly to 11.7% (95% CI 8.0 to 16.0%). Redefining the effectiveness calculation to include (incurable but treatable) herpes simplex virus (HSV)-2 ulcers, the overall effectiveness of syndromic STI treatment for ulcers was halved from 9.9% (95% CI 6.6 to 14.0%) to 4.1% (95% CI 2.7 to 5.8%). However, if HSV-2 antiviral treatment was included in the national guidelines and 90% of patients adhered, the overall effectiveness would increase to 9.9% (95% CI 6.7 to 13.9%).

### Trend in STI prevalences before and after the introduction of improved syndromic STI treatment services

The low effectiveness of the syndromic STI treatment services in rural KwaZulu-Natal estimated above is consistent with the trends in curable STI prevalences. Table 3 shows the data on gonorrhoea, chlamydia, trichomoniasis and syphilis prevalence from surveys of the general population and women attending antenatal, family planning and child immunisation clinics in this area. The available data related almost exclusively to antenatal clinic attendees. The black horizontal line shows the approximate timing of the introduction of syndromic STI treatment services in 1995.

**Table 3:** Summary of epidemiological data on classical STI prevalences from surveys of the general population and women attending antenatal, family planning and child immunisation clinics in rural northern KwaZulu-Natal, % (95% CI)

Data collection year and population characteristics	Mean age (range)	Sex	n	NG	CT	TV			Reference
							Serological TP (TPHA+/RPR⩾1:1)	High titre active TP (TPHA+/RPR⩾1:8)	
Median female prevalence (range):									
All		F		5.8 (4.0 to 10.0)	10.0 (6.4 to 13.5)		8.5 (2.0 to 11.9)		
1987–1995		F		5.7 (4.9 to 5.8)	8.9 (6.4 to 11.4)		11.9 (8.5 to 11.9)		
1996–2004		F		6.9 (4.0 to 10.0)	10.9 (7.4 to 13.5)	29.4 (15.8 to 36.8)	7.0 (2.0 to 8.5)	3.2 (2.0 to 3.6)	
1987, peripheral hospital ANC in Empangeni	26 (15 to 42)	F	193	5.7 (2.9 to 10.0)*	11.4 (7.3 to 16.7)†		11.9 (7.7 to 17.3)		15
1995, general population in R Hlabisa	28 SD = 9.4	M	90	2.3 (0.3 to 8.2)‡	5.6 (1.8 to 12.5)‡		9.3 (4.1 to 17.5)		16
		F	140	5.8 (2.6 to 11.2)‡	6.4 (3.0 to 11.9)‡		8.5 (4.4 to 14.3)		
1995, ANC in Hlabisa		F	327	4.9 (2.8 to 7.8)*	8.9 (6.0 to 12.5)†		11.9 (8.6 to 15.9)		Sturm, personal communication, 2005
1996, 4 ANCs (serving 1 TC, 1 PU, 2 R) in Hlabisa	25 SD = 6.6	F	271	7.8 (4.9 to 11.6)*	12.9 (9.2 to 17.5)†		8.4 (5.5 to 12.5)		17
1997§, Hlabisa hospital FP serving a TC	25 SD = 6.0	F	189	4.2 (1.8 to 8.2)*	7.4 (4.1 to 12.1)†		7.9 (4.5 to 12.8)		19
1998/00, all Hlabisa sub-district ANCs		F	474					3.6 (2.1 to 5.7)¶	18
1999, ANC at KwaMsane in Hlabisa sub-district, serving R & PU KzN	27 SD = 5.4	F	245	6.9 (4.1 to 10.9)**	11.0 (7.4 to 15.6)**	31.8 (26.1 to 38.1)††	6.1 (3.5 to 9.9)	2.0 (0.7 to 4.7)	Unpublished, Moodley, 2005
1999/00, ANC recruited for neonatal outcome follow-up at KwaMsane		F	650	10.0 (7.8 to 12.6)*	9.1 (7.0 to 11.6)†				Unpublished, Moodley, 2005
2002, ANC in Hlabisa		F	449	4.0 (2.4 to 6.3)**	10.9 (8.2 to 14.2)**	26.9 (22.9 to 31.3)††	2.0 (0.9 to 3.8)		20; Sturm, personal communication, 2005
2003, FP/IMM at 3 clinics in Hlabisa sub-district‡‡	29 (15–49)	F	346	5.6 (3.4 to 8.6)**	7.4 (4.8 to 10.7)**	15.8 (12.1 to 20.1)††		3.2 (1.6 to 5.7)	Unpublished, McGrath, 2005
2004§, ANC at KwaMsane PHC in Hlabisa sub-district	25 SD = 6.0	F	185	7.6 (4.2 to 12.4)§§	13.5 (8.9 to 19.3)¶¶	36.8 (29.8 to 44.1)***			21

Unadjusted for sample and diagnostic differences. Black horizontal line shows the timing of the introduction of syndromic STI treatment services.

*Culture on swabs; †immunofluoresence on swab ‡LCR on urine; §year published; ¶RPR⩾1:8 only, estimated from published data; **strand displacement assay; ††PCR; ‡‡a predominately HIV uninfected and low mobility population enrolled in microbicide feasibility study; §§culture and molecular methods on tampon and swab and urine; ¶¶molecular methods on tampon and swab and urine; ***culture and molecular methods on tampon and urine.

ANC, antenatal clinic; CT, *Chlamydia trachomatis*; F, female; FP, family planning clinic; HD, *Haemophilus ducreyi*; HSV-2, herpes simplex virus type 2; IMM, child immunisation clinic; KzN, KwaZulu-Natal; LGV, *Lympho-granuloma venereum*; M, male; NG, *Neisseria gonorrhoeae*; PU, peri-urban; R, rural; RPR, rapid plasma reagin; SD, standard deviation; STI, sexually transmitted infection; TC, trading centre; TP, *Treponema pallidum*; TPHA, *Treponema pallidum* haemoagglutination assay; TV, *Trichomonas vaginalis*.

The data show very high historic and current STI prevalences. Between 1987 and 2004, the median prevalences of gonorrhoea, chlamydia and serological syphilis were 5.8% (range 4.0–10.0%), 10.0% (range 6.4–13.5%) and 8.5% (range 2.0–11.9). The prevalences of gonorrhoea or chlamydia do not appear to have fallen since the introduction of syndromic STI treatment. Serological syphilis prevalence (*Treponema pallidum* haemagglutination assay (TPHA)+/RPR⩾1:1) may have fallen since the introduction of syndromic STI treatment. The median prevalence of serological syphilis was 11.9% (range 8.5–11.9%) prior to its introduction and 7.0% (range 2.0–8.5%) since. This fall in syphilis prevalence in rural northern KwaZulu-Natal is consistent with the rapid and sustained fall in the RPR titre among antenatal clinic attendees in the KwaZulu-Natal province, from 11% in 1997 to around 3% in 2001.^22^ Data were not available to explore trends for high-titre active syphilis or trichomoniasis.

## DISCUSSION

This study suggests that the syndromic STI treatment services in rural KwaZulu-Natal cure around 13% of symptomatic curable STI episodes. This low effectiveness is consistent with the continuing high prevalence of curable STIs in this population. Our results suggest that the effectiveness of curable syndromic STI treatment services could be improved most by increasing rates of treatment seeking and provision of the correct treatment.

Although our estimates were based on the best available data and robustly accounted for statistical uncertainty, some caution is required in the interpretation. Better data on some of the required parameter values would improve our estimates. For example, data on the quality of treatment provision were collected in the late 1990s and provision could have improved or deteriorated since then, which may lead to an underestimate or overestimate of effectiveness, respectively. The available data on STI trends suggest, however, that whatever the trend in overall effectiveness since the late 1990s, there is no evidence this has been accompanied by a marked change in STI rates. We had no information on the total number of syndrome episodes experienced by residents each year, and therefore our estimate of the annual number of symptomatic episodes in residents is likely to be a lower bound. We were also required to use cure rate data on ulcers and urethritis from an urban KwaZulu-Natal population, which may not a bovne be representative of the study area. However, the estimate of overall effectiveness was shown to be robust to increasing the cure rate to 100% and relatively insensitive to a reduction of 25% in this parameter value—a range likely to include the true value. Studies to collect more data on these parameters may be justified and could be used to update these estimates using the supplied WinBUGS code.

We may have overestimated overall effectiveness for at least two other reasons. If we overestimated the proportion of individuals seeking treatment at clinics or their GPs because, for example, we based our estimate on data from survey participants rather than all residents, or because patients actually went to traditional healers, then the overall effectiveness would be lower than estimated. Similarly, if the assumed cure rates were too high because, for example, they were based on data on patients followed-up rather than those lost to follow-up, then overall effectiveness would be lower than estimated. However, this effect was shown to be small and data collected in Tanzania in the mid-1990s showed little difference between cure rates among returners and non-returners.^26^

Conversely, we may have underestimated the overall effectiveness for at least two reasons. We assumed that the cure rate for incorrect treatment was 0%, but some treatments not in the national guidelines may be partially effective, increasing the effectiveness. Also, with the exception of the treatment for gonorrhoea, only the first-line treatment regimens were considered. Including the second-line treatment regimens would have increased effectiveness. However, as the assumed cure rates were already high, any underestimate will be small.

Our definition of overall effectiveness excluded asymptomatic STI episodes. This definition was chosen because it can be considered to be the upper limit for syndromic STI treatment (barring any indirect treatment via partner notification or cure of asymptomatic co-infections). However, as a large proportion of STI episodes are asymptomatic, particularly in women,^27^ even if all symptomatic episodes are cured a large proportion of STIs will remain untreated. This study could be extended to include asymptomatic episodes, but this would require currently unavailable aetiology-specific general population data on the frequency of STI episodes and the proportion of episodes that became symptomatic.

Our primary aim was to estimate the effectiveness of syndromic STI treatment services in treating curable STIs. However, the primary cause of genital ulcers in this population is HSV-2.^14^ HSV-2 infection is incurable and antiviral HSV-2 treatment is not recommended in the national guidelines.^6^ Our results suggest that if HSV-2 ulcers were included in the effectiveness calculation the proportion of ulcers cured or correctly treated was halved, but this reduction could be entirely countered by including episodic antiviral treatment in the national guidelines. The lack of treatment for HSV-2 ulcers may be an important reason for the low treatment seeking rates in this population, if individuals seeking treatment learn that treatment is ineffective. If true, then adding episodic HSV-2 antiviral treatment to the syndromic STI treatment guidelines may have the additional benefit of increasing the treatment seeking rates for other STIs.

Our STI prevalence trend analysis was limited by changes in diagnostic methods and sample populations over time and, therefore, statistical trend-tests were inappropriate. Molecular diagnostic methods, which have been replacing classical methods over the period under study, tend to have a higher sensitivity than classical methods and therefore it is possible that a decreasing trend in gonorrhoea and chlamydia may have been obscured. The contrasting fall in syphilis prevalence may be due, in part, to syphilis treatment of antenatal clinic attendees.^8^ Changes in sample populations over time and high rates of migration and urbanisation in the study area may also have led to under or overestimation of effectiveness.

A previous study estimated the proportion of women who were asymptomatic, symptomatic but not seeking care, and symptomatic and seeking care and infected with *T vaginalis, N gonorrhoeae, C trachomatis* or *T pallidum* on any given day in Hlabisa health district.^28^ In line with our findings, the authors concluded that STIs remained untreated because of low symptom recognition and treatment seeking rates. Our study updated this analysis, extended the work to include males, incorporated other ulcerative STIs, including HSV-2, and estimated the overall effectiveness of the syndromic STI treatment algorithm.

Another earlier study estimated the proportion of bacterial STI cured by primary healthcare services in Mwanza Region, Tanzania, before and after the introduction of improved STI treatment services, to be <10% and 23–41%, respectively.^29^ The higher effectiveness estimated for Mwanza than KwaZulu-Natal was primarily due to higher treatment seeking rates and higher rates of correct treatment. The higher rates in Tanzania may have been because community activities were more intensive as part of an intervention trial, or the division of STI treatment services between the private and public sectors in South Africa may make improving services more difficult than in Tanzania.

Only about 13% of symptomatic STI episodes may be cured by syndromic STI treatment services in rural KwaZulu-Natal and there is little evidence to show an improvement in the prevalence of curable STIs. As long as there is no acceptable alternative, syndromic STI treatment remains the only option for the management of STIs in the individual patient. As syndromic STI treatment may be a cost-saving HIV prevention strategy in this high HIV-incidence setting,^3^ innovative strategies are urgently needed to increase rates of treatment seeking and the provision of the correct treatment.

**Key messages**

- Overall effectiveness of syndromic treatment for curable sexually transmitted infections (STIs) in rural KwaZulu-Natal remains low (13.1%, 95% CI 8.9 to 17.8% of symptomatic curable STI episodes cured) and there is little evidence of reduced curable STI prevalences.
- As syndromic treatment is likely to be a cost-saving HIV prevention intervention in South Africa, innovative strategies are urgently needed to increase rates of treatment seeking and correct treatment provision.
- The model used to make this estimate is provided in full in the online material and could be used to (re)estimate effectiveness in this and other settings.
